# Vitamin D binding protein genetic isoforms, serum vitamin D, and cancer risk in the Prostate, Lung, Colorectal, and Ovarian (PLCO) Cancer Screening Trial

**DOI:** 10.1371/journal.pone.0315252

**Published:** 2024-12-20

**Authors:** Stephanie J. Weinstein, Dominick Parisi, Alison M. Mondul, Tracy M. Layne, Jiaqi Huang, Rachael Z. Stolzenberg-Solomon, Regina G. Ziegler, Mark P. Purdue, Wen-Yi Huang, Christian C. Abnet, Neal D. Freedman, Kai Yu, Demetrius Albanes

**Affiliations:** 1 Division of Cancer Epidemiology and Genetics, National Cancer Institute, NIH, Bethesda, Maryland, United States of America; 2 Information Management Services, Inc., Calverton, Maryland, United States of America; 3 Department of Epidemiology, University of Michigan School of Public Health, Ann Arbor, Michigan, United States of America; 4 Icahn School of Medicine at Mount Sinai, New York, New York, United States of America; 5 Department of Metabolism and Endocrinology, The Second Xiangya Hospital of Central South University, Changsha, Hunan, China; 6 Division of Cancer Control and Population Sciences, National Cancer Institute, NIH, Bethesda, Maryland, United States of America; Medical University of Gdańsk, POLAND

## Abstract

Associations between vitamin D biochemical status and cancer may be modified by vitamin D binding protein isoforms which are encoded by *GC* (group-specific component). We examined interactions between serum 25-hydroxyvitamin D [25(OH)D], the Gc isoforms Gc1-1, Gc1-2, and Gc2-2, and cancer risk within the Prostate, Lung, Colorectal, and Ovarian Cancer Screening Trial cohort based on 3,795 cases and 3,856 controls. Multivariable-adjusted logistic regression models estimated odds ratios (ORs) and 95% confidence intervals (CIs) of cancer risk according to 25(OH)D quantiles, stratified by Gc isoform. Separately, the *GC*-cancer risk association was examined using proportional hazards regression among 109,746 individuals with genetic data and 26,713 diagnosed with cancer. Specific vitamin D binding protein isoform subtypes were delineated and analyzed, including Gc1-1 subtypes (Gc1s-Gc1s, Gc1f-Gc1s, and Gc1f-Gc1f) and Gc2 subtypes (Gc1s-Gc2, Gc1f-Gc2, and Gc2-Gc2). For most cancers, the *GC* genotype did not modify the risk associations for 25(OH)D; e.g., the OR for high vs. low vitamin D quintile was 1.09 (0.89–1.33) for overall cancer risk among individuals with the Gc1-1 isoform and 1.04 (0.83–1.31) among those with either the Gc1-2 or Gc2-2 isoforms. ORs for high compared to low vitamin D tertile for colorectal, lung, breast, and prostate cancer among those with the Gc1-1 vs. any Gc2 isoforms were, respectively, 0.60 vs. 0.73, 1.96 vs. 1.03, 1.30 vs. 1.18, and 1.19 vs. 1.22 (all p-interaction ≥0.36). However, *GC* qualitatively modified the vitamin D-bladder cancer risk association: OR = 1.70 (95% CI 0.96–2.98) among those with the Gc1-1 isoform and 0.52 (0.28–0.96) among those with any Gc2 isoforms (p-interaction = 0.03). When modeled without regard for 25(OH)D, Gc isoforms were generally not associated with cancer risk, although melanoma risk was significantly lower among individuals with the “f” subtype of the Gc1-1 isoform, specifically HR = 0.83 (95% CI 0.70–0.98) for Gc1f-1s and 0.67 (0.45–1.00) for Gc1f-1f, compared to individuals with the Gc1s-Gc1s isoform. Vitamin D binding protein genetic isoforms may be associated with melanoma risk but do not modify the association between vitamin D status and cancer, with the possible exception of bladder cancer.

## Introduction

Vitamin D has been extensively examined in regard to cancer risk. Studies suggest that higher circulating 25-hydroxyvitamin concentrations [25(OH)D, the accepted biomarker of vitamin D status] are associated with lower risk of colorectal cancer [1], but not breast [2] or lung cancer [3], or cancer at other sites [4–9], and may be associated with higher risk of prostate cancer [10]. Vitamin D binding protein is responsible for the transport of vitamin D and its metabolites through the circulation [11], and two single nucleotide polymorphisms (SNPs), rs7041 and rs4588 [12], define three isoforms: Gc1s, Gc1f, and Gc2. (Vitamin D binding protein was originally known as “group-specific component” or Gc, the acronym its encoding gene retains.) The affinity of vitamin D binding protein for 25(OH)D and 1,25-dihydroxyvitamin D [1,25(OH)_2_D, the active form of vitamin D] varies by isoform, with Gc1f having the strongest affinity, Gc2 having the weakest, and Gc1s having intermediate affinity [11]. This results in differences in actual concentrations of 25(OH)D and 1,25(OH)_2_D, with highest concentrations found for Gc1-1, intermediate for Gc1-2, and lowest for Gc2-2 [13, 14]. We recently showed in the Prostate, Lung, Colorectal, and Ovarian (PLCO) Cancer Screening Trial that associations between vitamin D and both overall cancer survival and lung cancer survival were modified by Gc isoforms such that circulating vitamin D was associated with increased survival only for those cases with the Gc2 isoform [15]. A similar pattern was noted by Gibbs *et al*. for colorectal adenoma and cancer risk, as well as for colorectal cancer mortality [16–18] and for vitamin D supplementation and colorectal adenoma recurrence [19], and by another group for vitamin D intake and colorectal cancer risk [20]. However, data from three other studies did not find effect modification of the vitamin D-cancer association by Gc isoform for colorectal [21, 22] or breast cancer [12], and associations with other sites are unknown. Therefore, the rationale for this analysis was to examine the genetic influence of *GC*, which encodes the vitamin D binding protein, on the risk of cancer overall and multiple specific organ sites, and to further assess whether *GC* acts as an effect modifier of the relationship between circulating vitamin D and overall and site-specific cancer risk. Accordingly, we examined whether Gc isoforms modify the association between serum 25(OH)D and site-specific and overall cancer risk within the PLCO Trial.

## Materials and methods

### Study population

The PLCO Trial was a randomized trial of prostate, lung, colorectal, and ovarian cancer screening conducted within 10 centers in the United States from November 16, 1993 to July 2, 2001. Men and women (n = 154,987) aged 55–74 years completed a self-administered questionnaire at baseline, which gathered information on sociodemographic and risk factor characteristics including age, race, ethnicity, height, weight, smoking history, family history of cancer, and personal medical history. Non-fasting serum samples were collected and stored at –70°C. Written informed consent was obtained from each participant and the PLCO Trial was approved by the Institutional Review Boards of the National Cancer Institute and the 10 PLCO centers [23–25].

Only participants with available genetic data (n = 109,746) were included in this analysis. Of these, 26,713 had a cancer diagnosis during follow-up. The majority of the analyses conducted here used a subset of participants (n = 7,651) with both available genetic data and previously measured circulating vitamin D in nested matched case-control sets [3–6, 8, 9, 24, 26–30]. These included the following cancer sites, listed here first with their total number of cases and then the number of cases with both available circulating vitamin D and genotype data: biliary (195/NA), bladder (1,223/342), breast (4,013/862), male breast (40/NA), colorectum (2,322/416), endometrium (838/135), glioma (306/NA), hematopoietic cancer (3,660/278), head and neck (689/NA), kidney (983/145), liver (305/NA), lung (3,394/389), melanoma (1,389/NA), ovary (515/58), pancreas (865/194), prostate (8282/912), thyroid (380/NA), and upper gastrointestinal tract (646/75).

### Cancer diagnoses and control selection

Incident cancer cases were identified through 2017 using a variety of methods including linkage to state cancer registries, physician reports, death certificates, self-report, and/or next-of-kin report. Trained medical record specialists confirmed the diagnoses with medical record abstractions. For the vitamin D nested sets, censor dates for cancer diagnoses varied, as listed in the original reports [3–6, 8, 9, 24, 26–31].

All nested case-control sets were matched on age (+/- 1 year, +/- 5 years, or 5-year intervals) and sex (or were only one sex). For race, one prostate case-control set included only non-Hispanic white participants [24] and another included only non-Hispanic black participants (1:2 case:control ratio) [27]. All but two [3, 26] of the remaining studies were matched on race, and all but two [24, 26] were matched on date of blood collection (+/- 30 days or 2-month blocks).

### Vitamin D status

Circulating 25(OH)D was measured using radioimmunoassay (for breast, prostate among non-Hispanic white individuals, and 76% of the pancreas set) [32, 33], liquid chromatography/mass spectrometry (for prostate among non-Hispanic black individuals and lung) [27, 34] and chemiluminescence immunoassay (all other case-control sets including the remaining pancreas set) [35]. Coefficients of variation ranged from 3.7%-13.2% [3–6, 8, 9, 24, 26–31].

### Genotyping

Genotyping was conducted using Illumina arrays and imputed against the TopMed reference panel 5b through the Michigan Imputation Server. All imputed genotypes were converted to the bgen v1.2 format with plink2. Imputed genotypes (range 0–2) for the vitamin D binding protein SNPs rs7041 and rs4588 were converted to categorical variables (i.e., 0, 1, 2) using cutpoints <0.15, >0.85 & <1.15, and >1.85, respectively. Values outside these ranges were excluded and only participants with data for both SNPs were used in the analyses. S1 Table displays how cross tabulations of rs4588 and rs7041 define the six vitamin D binding protein Gc isoforms (Gc1s-Gc1s, Gc1f-Gc1s, Gc1f-Gc1f, Gc1s-Gc2, Gc1f-Gc2, and Gc2-Gc2.) Empty cells indicate rare combinations not found in our data. Across the rows, “Gc1-1” includes isoforms with only Gc1 (i.e., Gc1s-Gc1s, Gc1f-Gc1s, or Gc1f-Gc1f), “Gc1-2” includes Gc1s-Gc2 and Gc1f-Gc2 and “Gc2” includes only Gc2-2. The term “any Gc2” means having any Gc2 isoform, i.e., Gc1s-Gc2, Gc1f-Gc2, or Gc2-Gc2. Results for the gene-only analyses with over 100,000 participants are presented for each of the six isoforms separately, while results for the analyses using the vitamin D data (n = 7,651) are presented with the isoforms collapsed as Gc1-1, Gc1-2, and Gc2-2 because of the smaller number.

### Statistical analysis

For analyses among the 109,746 participants with *GC* SNP data, cases were defined based on the 2017 cancer incidence censor date. For analyses including circulating 25(OH)D data, cases and controls were defined as they were when originally selected for each nested case-control set. Characteristics of cancer cases vs. controls were compared using Wilcoxon rank sum tests (for continuous variables) and chi-square tests (for categorical variables). Unconditional logistic regression was used to calculate odds ratios (ORs) and 95% confidence intervals (CIs) for vitamin D quantiles, stratified by *GC* SNP, and adjusted for factors potentially associated with cancer risk including age at randomization, sex, race, body mass index (BMI), smoking status, physical activity, history of diabetes, and family history of cancer. The lung cancer models were additionally adjusted for cigar smoking, pipe smoking, and packyears of cigarette smoking. Using separate smoking duration and intensity variables in the lung cancer model or adding the smoking-related covariates to the overall cancer models did not materially change the risk estimates. The breast, endometrial, and ovarian cancer models were additionally adjusted for parity, duration of oral contraceptive use, and menopausal status/duration of menopausal hormone use, but these covariate adjustments did not materially change the risk estimates and were therefore not retained. Vitamin D quintiles were calculated separately for each cancer site by sex and season (December–May vs. June–November) and merged for the overall cancer model. For the organ-specific models, due to small samples sizes in the Gc2 strata, vitamin D was categorized into tertiles and Gc1-2 and Gc2-2 groups were combined to produce more stable risk estimates. See S2 and S3 Tables for quintile and tertile cut points, respectively. Persons with missing BMI were assigned the median BMI value and missing codes were included for categorical variables.

Tests for linear trend were conducted by coding each category either 1–5 (for quintiles) or 1–3 (for tertiles) and treating these as continuous variables. The log-likelihood test was used to statistically evaluate effect modification of the Gc isoform on the vitamin D/cancer association, by comparing models with and without a cross-product term of the vitamin D quantiles (n = 5 or n = 3) and the Gc isoform (n = 2 or n = 3 categories).

Cox proportional hazards regression was used to calculate hazard ratios (HRs) and 95% CIs to examine the association between the Gc SNPs and cancer risk, without regard to circulating vitamin D (total n = 109,746, n with cancer = 26,713). Models were adjusted for the same factors as above, except for physical activity which was only ascertained in the screening arm of the trial. Because 2.8% of the participants had more than one cancer diagnosis, the follow-up time for these participants was censored at the date of the first cancer diagnosis to account for competing risks; however, this did not change the results compared with censoring at the date of the cancer diagnosis for each particular cancer site model (whether or not it was the first cancer diagnosis).

Statistical analyses were conducted using SAS version 9.4 (SAS Institute, Inc., Cary, North Carolina) and all *P*-values were 2-sided. The data were last accessed on November 30, 2023.

## Results

Cancer cases were significantly older at randomization than controls and more likely to be current or former smokers (Table 1). The cases also included a lower percentage of Black individuals compared with controls owing to the 2:1 control:case ratio in that study set. Median serum 25(OH)D was similar in cases compared with controls, as was the distribution of the Gc isoform (Table 1). As expected, the distribution of Gc isoform differs by race, with the Gc1f isoform being more common in persons of African ancestry (S4 Table). Within the entire PLCO dataset with *GC* SNP data, cancer cases were more likely to be older, male, current smokers, and to have reported a family history of cancer (S5 Table). The distribution of Gc isoform did not differ by case status.

**Table 1:** Selected baseline characteristics of cases and controls with circulating 25(OH)D and Gc genotype data.

Characteristic	Cancer cases (n = 3,795)	Controls (n = 3,856)	*P* ^a^
Age at randomization, years (median, 10^th^-90^th^)	64 (57–71)	63 (57–71)	0.01
Sex, No. (%)			0.84
Female	1,670 (44.0)	1,688 (43.8)	
Male	2,125 (56.0)	2,168 (56.2)	
Race, No. (%)			<0.0001
American Indian individuals	5 (0.1)	6 (0.2)	
Asian individuals	67 (1.8)	86 (2.2)	
Black individuals, non-Hispanic	333 (8.8)	489 (12.7)	
Hispanic individuals	18 (0.5)	29 (0.8)	
Pacific Islander individuals	4 (0.1)	7 (0.2)	
White individuals, non-Hispanic	3,368 (88.8)	3,237 (84.0)	
Missing	0	2 (0.1)	
BMI, kg/m^2^ (median, 10^th^-90^th^)	26.6 (22.3–33.5)	26.6 (22.1–33.4)	0.15
Smoking status, No. (%)			0.02
Never	1,557 (41.0)	1,715 (44.5)	
Former	1,803 (47.5)	1,718 (44.6)	
Current	434 (11.4)	421 (10.9)	
Vigorous physical activity, No. (%)			0.94
None or <1 hours/week	1,170 (30.8)	1,192 (30.9)	
1–2 hours/week	1,002 (26.4)	998 (25.9)	
3+ hours/week	1,372 (36.2)	1,401 (36.3)	
Missing	251 (6.6)	265 (6.9)	
History of diabetes, No. (% yes)	309 (8.1)	319 (8.3)	0.27
Family history of cancer, No. (% yes)^b^	2,201 (58.0)	2,154 (55.9)	0.15
Winter blood collection (Dec-May), No. (%)	1,841 (48.5)	1,863 (48.3)	0.86
Serum 25(OH)D, nmol/L (median, 10^th^-90^th^)	58.0 (31.7–87.6)	56.9 (31.4–88.4)	0.33
Latitude of study center, No. (%)			0.04
<34°N	258 (6.8)	306 (7.9)	
34-<42°N	1,846 (48.6)	1,781 (46.2)	
*≥*42°N	1,691 (44.6)	1,769 (45.9)	
Gc isoform, No. (%)			0.46
Gc1-Gc1	2,076 (54.7)	2,097 (54.4)	
Gc1-Gc2	1,423 (37.5)	1,483 (38.5)	
Gc2-Gc2	296 (7.8)	276 (7.2)	

BMI = body mass index; 25(OH)D, 25-hydroxyvitamin D

^a^ *P*-values based on 2-sided Wilcoxon rank sum tests for continuous variables and 2-sided chi-square tests for categorical variables

^b^ Family history of any cancer reported in first degree relatives (i.e. parents, full-siblings, and children)

Circulating vitamin D was not associated with overall cancer, although risk was suggestively elevated in quintiles three through five vs. quintile one, with a significant trend test (p-trend = 0.04; Table 2). Risk of overall cancer was significantly elevated in quintile four for individuals with any Gc-2, with a marginally significant trend (p-trend = 0.06), but the statistical test for a 25(OH)D-Gc isoform interaction was not significant (p-interaction = 0.46; Table 2).

**Table 2:** Association between prediagnostic serum 25(OH)D and overall cancer risk, stratified by Gc isoform^a^.

	Season-, sex-, and cancer site-specific quintile of serum 25(OH)D^b^					p-trend^c^
	1	2	3	4	5	
Overall cancer						
Number of cases/controls	729/790	689/777	811/767	810/775	756/747	
Multivariable-adjusted OR (95% CI)	1.00 (Referent)	0.95 (0.82–1.09)	1.13 (0.98–1.30)	1.12 (0.97–1.29)	1.10 (0.95–1.28)	0.04
Stratified by Gc isoform^d^						
Gc1-1 (2076 cases/2097 controls)						
Number of cases/controls	349/376	355/379	420/401	453/469	499/472	
Multivariable-adjusted OR (95% CI)	1.00 (Referent)	0.96 (0.78–1.18)	1.06 (0.86–1.30)	0.98 (0.80–1.20)	1.09 (0.89–1.33)	0.41
Gc1-2 (1423 cases/1483 controls)						
Number of cases/controls	303/332	272/328	316/309	300/262	232/252	
Multivariable-adjusted OR (95% CI)	1.00 (Referent)	0.90 (0.72–1.13)	1.11 (0.89–1.40)	1.27 (1.01–1.61)	1.03 (0.80–1.31)	0.12
Gc2-2 (296 cases/276 controls)						
Number of cases/controls	77/82	62/70	75/57	57/44	25/23	
Multivariable-adjusted OR (95% CI)	1.00 (Referent)	0.98 (0.61–1.57)	1.47 (0.91–2.37)	1.47 (0.88–2.47)	1.20 (0.62–2.34)	0.11
Any Gc-2 (Gc1-2 and Gc2-2 categories combined, 1719 cases/1759 controls)						
Number of cases/controls	380/414	334/398	391/366	357/306	257/275	
Multivariable-adjusted OR (95% CI)	1.00 (Referent)	0.91 (0.74–1.12)	1.16 (0.95–1.42)	1.29 (1.04–1.59)	1.04 (0.83–1.31)	0.06

^a^ Adjusted for age at randomization (continuous), race (non-Hispanic white, non-Hispanic black, all others combined), sex, body mass index (continuous), smoking status (never, current, former), vigorous physical activity (none or <1 hour/week, 1–2 hours/week, 3+ hours/week), history of diabetes (yes/no), family history of cancer (yes/no), latitude of study center (<34°N, 34-<42°N, >42°N)

^b^ Cut-points for the 25(OH)D concentrations (nmol/L) were based on the distribution by season and sex for each cancer site and are listed in S2 Table

^c^Tests for linear trend were conducted by coding each quintile category as 1–5 and treating this as a continuous variable

^d^p-interaction = 0.46

For colorectal cancer, we observed the expected pattern of reduced risk with higher 25(OH)D, but this did not differ significantly by Gc isoform (p-interaction = 0.92; Table 3). Risks between circulating 25(OH)D and breast and prostate cancer also did not differ by Gc isoform (p-interaction = 0.41 and 0.36, respectively), whereas lung cancer risk was significantly higher for elevated vitamin D among individuals with the Gc1-1 isoform (OR for highest vs. lowest tertile of vitamin D = 1.96, 95% CI 1.14–3.37, p-trend = 0.01), but not for those with the Gc1-2 or Gc2-2 isoform (OR = 1.03, 95% CI 0.60–1.80, p-trend = 0.90), although the interaction was not significant (p = 0.45). The Gc isoform did modify the association between circulating vitamin D and bladder cancer risk: OR’s for the highest vs. lowest tertile of vitamin D were 1.70, 95% CI 0.96–2.98 (p-trend = 0.07) for individuals with the Gc1 isoform and 0.52, 95% CI 0.28–0.96 (p-trend = 0.04) for individuals with the Gc1-2 or Gc2-2 isoforms (p-interaction = 0.03). Vitamin D was not associated with overall bladder cancer risk, however, with OR (95% CI) 1.11 (0.76–1.62) and 1.00 (0.67–1.50) for 25(OH)D tertiles 2 and 3, respectively (vs. tertile 1). Risks did not differ by Gc isoform for other cancer sites.

**Table 3:** Association between prediagnostic serum 25(OH)D and risk of site-specific cancers, stratified by Gc isoform^a^.

Cancer site	Season-, sex-, and cancer site-specific tertile of serum 25(OH)D^b^				
	1	2	3	p-trend^c^	p-interaction
**Bladder** Gc1-1 (191 cases/182 controls)					0.03
Number of cases/controls	47/61	67/59	77/62		
Multivariable-adjusted OR (95% CI)	1.00 (Referent)	1.41 (0.81–2.45)	1.70 (0.96–2.98)	0.07	
Gc1-2 and Gc2-2 categories combined (151 cases/170 controls)					
Number of cases/controls	63/59	56/59	32/52		
Multivariable-adjusted OR (95% CI)	1.00 (Referent)	0.82 (0.47–1.43)	0.52 (0.28–0.96)	0.04	
**Breast** Gc1-1 (429 cases/460 controls)					0.41
Number of cases/controls	99/130	158/143	172/187		
Multivariable-adjusted OR (95% CI)	1.00 (Referent)	1.54 (1.07–2.22)	1.30 (0.90–1.86)	0.29	
Gc1-2 and Gc2-2 categories combined (433 cases/441 controls)					
Number of cases/controls	156/170	168/161	109/110		
Multivariable-adjusted OR (95% CI)	1.00 (Referent)	1.24 (0.90–1.70)	1.18 (0.81–1.71)	0.33	
**Colorectum** Gc1-1 (234 cases/255 controls)					0.92
Number of cases/controls	86/82	85/83	63/90		
Multivariable-adjusted OR (95% CI)	1.00 (Referent)	0.94 (0.59–1.49)	0.60 (0.37–0.98)	0.04	
Gc1-2 and Gc2-2 categories combined (182 cases/198 controls)					
Number of cases/controls	68/71	74/69	40/58		
Multivariable-adjusted OR (95% CI)	1.00 (Referent)	1.16 (0.71–1.90)	0.73 (0.41–1.28)	0.33	
**Endometrium** Gc1-1 (73 cases/61 controls)					0.61
Number of cases/controls	24/21	23/19	26/21		
Multivariable-adjusted OR (95% CI)	1.00 (Referent)	1.34 (0.51–3.54)	2.08 (0.76–5.71)	0.15	
Gc1-2 and Gc2-2 categories combined (62 cases/78 controls)					
Number of cases/controls	23/26	24/28	15/24		
Multivariable-adjusted OR (95% CI)	1.00 (Referent)	1.04 (0.44–2.49)	0.71 (0.28–1.77)	0.48	
**Hematopoietic** Gc1-1 (145 cases/137 controls)					0.48
Number of cases/controls	32/34	47/55	66/48		
Multivariable-adjusted OR (95% CI)	1.00 (Referent)	0.97 (0.50–1.90)	1.95 (0.97–3.91)	0.03	
Gc1-2 and Gc2-2 categories combined (133 cases/148 controls)					
Number of cases/controls	41/63	38/42	54/43		
Multivariable-adjusted OR (95% CI)	1.00 (Referent)	1.56 (0.85–2.88)	2.13 (1.17–3.88)	0.01	
**Kidney** Gc1-1 (81 cases/87 controls)					0.18
Number of cases/controls	34/26	22/31	25/30		
Multivariable-adjusted OR (95% CI)	1.00 (Referent)	0.62 (0.27–1.43)	0.72 (0.32–1.61)	0.44	
Gc1-2 and Gc2-2 categories combined (64 cases/65 controls)					
Number of cases/controls	22/27	19/21	23/17		
Multivariable-adjusted OR (95% CI)	1.00 (Referent)	1.05 (0.38–2.90)	1.70 (0.61–4.77)	0.32	
**Lung**^**d**^ Gc1-1 (196 cases/224 controls)					0.45
Number of cases/controls	55/71	56/68	85/85		
Multivariable-adjusted OR (95% CI)	1.00 (Referent)	1.35 (0.77–2.39)	1.96 (1.14–3.37)	0.01	
Gc1-2 and Gc2-2 categories combined (193 cases/194 controls)					
Number of cases/controls	68/64	66/77	59/53		
Multivariable-adjusted OR (95% CI)	1.00 (Referent)	0.74 (0.44–1.24)	1.03 (0.60–1.80)	0.95	
**Ovary** Gc1-1 (31 cases/40 controls)					0.99
Number of cases/controls	10/15	9/13	12/12		
Multivariable-adjusted OR (95% CI)	1.00 (Referent)	0.65 (0.14–2.95)	0.74 (0.18–3.00)	0.68	
Gc1-2 and Gc2-2 categories combined (27 cases/29 controls)					
Number of cases/controls	10/9	7/10	10/10		
Multivariable-adjusted OR (95% CI)	1.00 (Referent)	0.94 (0.19–4.67)	1.01 (0.22–4.78)	0.98	
**Pancreas** Gc1-1 (107 cases/221 controls)					0.24
Number of cases/controls	45/73	29/67	33/81		
Multivariable-adjusted OR (95% CI)	1.00 (Referent)	0.84 (0.45–1.57)	0.77 (0.41–1.44)	0.41	
Gc1-2 and Gc2-2 categories combined (87 cases/179 controls)					
Number of cases/controls	36/62	24/70	27/47		
Multivariable-adjusted OR (95% CI)	1.00 (Referent)	0.55 (0.28–1.09)	1.03 (0.50–2.13)	0.99	
**Prostate** Gc1-1 (554 cases/661 controls)					0.36
Number of cases/controls	137/198	193/229	224/234		
Multivariable-adjusted OR (95% CI)	1.00 (Referent)	1.10 (0.82–1.49)	1.19 (0.88–1.61)	0.25	
Gc1-2 and Gc2-2 categories combined (358 cases/424 controls)					
Number of cases/controls	116/168	138/133	104/123		
Multivariable-adjusted OR (95% CI)	1.00 (Referent)	1.44 (1.01–2.05)	1.22 (0.84–1.77)	0.26	
**Upper gastrointestinal tract** Gc1-1 (39 cases/42 controls)					0.66
Number of cases/controls	11/12	11/14	17/16		
Multivariable-adjusted OR (95% CI)	1.00 (Referent)	1.23 (0.33–4.62)	1.65 (0.40–6.78)	0.49	
Gc1-2 and Gc2-2 categories combined (36 cases/49 controls)					
Number of cases/controls	11/22	15/16	10/11		
Multivariable-adjusted OR (95% CI)	1.00 (Referent)	2.70 (0.80–9.03)	2.21 (0.61–7.92)	0.19	

^a^ Adjusted for age at randomization (continuous), race (non-Hispanic white, non-Hispanic black, all others combined), sex, body mass index (continuous), smoking status (never, current, former), vigorous physical activity (none or <1 hour/week, 1–2 hours/week, 3+ hours/week), history of diabetes (yes/no), family history of cancer (yes/no), latitude of study center (<34°N, 34-<42°N, >42°N)

^b^ Cutpoints for the 25(OH)D concentrations (nmol/L) were based on the distribution by season and sex for each cancer site and are listed in S3 Table

^c^ Tests for linear trend were conducted by coding each tertile category as 1–3 and treating these as continuous variables

^d^ lung cancer model additionally adjusted for cigar smoking (yes/no), pipe smoking (yes/no), and packyears of cigarette smoking (quartiles)

Compared to individuals with the Gc1s-Gc1s isoform, those with other isoforms (Gc1f-Gc1s, Gc1f-Gc1f, Gc1s-Gc2, Gc1f-Gc2, Gc2-Gc2) experienced similar risks of overall and site-specific cancer, with HRs for overall cancer ranging from 0.96–0.99 (Table 4). An increased risk of biliary tract cancer was noted for individuals with the Gc2-Gc2 isoform (HR = 1.67, 95% CI 1.00–2.78) and an increased risk for breast cancer was noted for men with the Gc1f-Gc1s or Gc1f-Gc1f isoforms (HR = 2.52, 95% CI 1.02–6.20 and HR = 3.75, 95% CI = 1.06–13.29, respectively), but these were based on a small number of incident cases. We observed a reduced risk of melanoma for individuals with the Gc1f-Gc1s or Gc1f-Gc1f isoforms (HR = 0.83, 95% CI 0.70–0.98 and HR = 0.67, 95% CI 0.45–1.00, respectively). While we noted above that the Gc isoforms modified the vitamin D-bladder cancer association, there were no direct associations between the Gc isoforms and bladder cancer risk. When restricting the analysis to Black individuals, the various isoforms were also not associated with risk of overall cancer or any specific cancer site examined (S6 Table). Many HRs had wide CIs and several could not be calculated due to small numbers.

**Table 4:** Association between Gc isoform and overall and organ-specific cancer risk in 109,746 PLCO participants^a^.

Cancer site	Gc Isoform					
	Gc1s-Gc1s	Gc1f-Gc1s	Gc1f-Gc1f	Gc1s-Gc2	Gc1f-Gc2	Gc2-Gc2
Overall Number of cases/controls	8199/24904	4688/14686	1311/4056	8056/25174	2442/7750	2017/6463
Multivariable-adjusted HR (95% CI)	1.00 (Referent)	0.98 (0.94–1.01)	0.99 (0.93–1.06)	0.98 (0.95–1.01)	0.98 (0.93–1.02)	0.96 (0.92–1.01)
Biliary tract Number of cases/controls	49/33054	33/19341	9/5358	63/33167	20/10172	21/8459
Multivariable-adjusted HR (95% CI)	1.00 (Referent)	1.14 (0.73–1.77)	1.10 (0.50–2.42)	1.28 (0.88–1.86)	1.29 (0.76–2.19)	1.67 (1.00–2.78)
Bladder Number of cases/controls	384/32719	211/19163	45/5322	391/32839	100/10092	92/8388
Multivariable-adjusted HR (95% CI)	1.00 (Referent)	0.97 (0.82–1.14)	0.88 (0.63–1.24)	1.02 (0.89–1.18)	0.90 (0.72–1.12)	0.96 (0.76–1.20)
Breast (female) Number of cases/controls	1251/31852	699/18675	170/5197	1204/32026	365/9827	324/8156
Multivariable-adjusted HR (95% CI)	1.00 (Referent)	0.98 (0.89–1.08)	0.95 (0.80–1.14)	0.96 (0.88–1.04)	0.96 (0.85–1.08)	1.00 (0.88–1.13)
Breast (male) Number of cases/controls	8/33095	12/19362	5/5362	8/33222	4/10188	3/8477
Multivariable-adjusted HR (95% CI)	1.00 (Referent)	2.52 (1.02–6.20)	3.75 (1.06–13.29)	1.00 (0.38–2.66)	1.62 (0.48–5.44)	1.51 (0.40–5.68)
Colorectum Number of cases/controls	674/32429	398/18976	133/5234	710/32520	222/9970	185/8295
Multivariable-adjusted HR (95% CI)	1.00 (Referent)	1.01 (0.89–1.14)	1.18 (0.95–1.46)	1.05 (0.95–1.17)	1.08 (0.92–1.25)	1.08 (0.92–1.27)
Colon Number of cases/controls	560/32429	331/18976	114/5234	587/32520	189/9970	149/8295
Multivariable-adjusted HR (95% CI)	1.00 (Referent)	1.01 (0.88–1.16)	1.22 (0.97–1.54)	1.05 (0.93–1.18)	1.11 (0.94–1.31)	1.05 (0.88–1.26)
Rectum Number of cases/controls	103/32429	55/18976	13/5234	108/32520	28/9970	25/8295
Multivariable-adjusted HR (95% CI)	1.00 (Referent)	0.93 (0.67–1.29)	0.86 (0.45–1.63)	1.05 (0.80–1.37)	0.91 (0.60–1.39)	0.96 (0.62–1.49)
Endometrium Number of cases/controls	260/32843	161/19213	24/5343	250/32980	76/10116	67/8413
Multivariable-adjusted HR (95% CI)	1.00 (Referent)	1.13 (0.93–1.38)	0.85 (0.55–1.33)	0.97 (0.81–1.15)	1.03 (0.79–1.33)	1.01 (0.77–1.33)
Glioma Number of cases/controls	96/33007	64/19310	8/5359	86/33144	30/10162	22/8458
Multivariable-adjusted HR (95% CI)	1.00 (Referent)	1.19 (0.86–1.63)	0.67 (0.31–1.45)	0.90 (0.67–1.20)	1.09 (0.72–1.64)	0.90 (0.57–1.43)
Hematopoietic Number of cases/controls	1159/31944	653/18721	130/5237	1136/32094	302/9890	280/8200
Multivariable-adjusted HR (95% CI)	1.00 (Referent)	1.00 (0.91–1.10)	0.88 (0.72–1.07)	0.98 (0.90–1.07)	0.91 (0.80–1.03)	0.96 (0.84–1.09)
Head and Neck Number of cases/controls	220/32883	113/19261	26/5341	210/33020	67/10125	53/8427
Multivariable-adjusted HR (95% CI)	1.00 (Referent)	0.87 (0.69–1.10)	0.71 (0.45–1.11)	0.95 (0.79–1.15)	0.99 (0.75–1.30)	0.95 (0.70–1.28)
Kidney Number of cases/controls	295/32808	178/19196	63/5304	284/32946	84/10108	79/8401
Multivariable-adjusted HR (95% CI)	1.00 (Referent)	1.02 (0.85–1.24)	1.25 (0.91–1.71)	0.96 (0.82–1.13)	0.93 (0.73–1.19)	1.05 (0.82–1.35)
Liver Number of cases/controls	78/33025	59/19315	20/5347	98/33132	29/10163	21/8459
Multivariable-adjusted HR (95% CI)	1.00 (Referent)	1.19 (0.85–1.68)	1.12 (0.64–1.96)	1.26 (0.93–1.69)	1.07 (0.69–1.65)	1.03 (0.64–1.67)
Lung ^b^ Number of cases/controls	1035/32068	613/18761	184/5183	1004/32226	318/9874	240/8240
Multivariable-adjusted HR (95% CI)	1.00 (Referent)	0.98 (0.88–1.08)	0.88 (0.74–1.06)	0.97 (0.86–1.05)	0.97 (0.86–1.11)	0.94 (0.82–1.09)
Melanoma Number of cases/controls	470/32633	210/19164	26/5341	440/32790	134/10058	109/8371
Multivariable-adjusted HR (95% CI)	1.00 (Referent)	0.83 (0.70–0.98)	0.67 (0.45–1.00)	0.94 (0.82–1.07)	1.07 (0.88–1.30)	0.93 (0.75–1.14)
Ovary Number of cases/controls	160/32943	91/19283	20/5347	163/33067	45/10147	36/8444
Multivariable-adjusted HR (95% CI)	1.00 (Referent)	1.00 (0.77–1.30)	0.87 (0.52–1.46)	1.01 (0.81–1.26)	0.93 (0.66–1.29)	0.87 (0.61–1.25)
Pancreas Number of cases/controls	251/32852	152/19222	47/5320	278/32952	79/10113	58/8422
Multivariable-adjusted HR (95% CI)	1.00 (Referent)	1.00 (0.82–1.23)	0.96 (0.67–1.36)	1.10 (0.93–1.31)	0.97 (0.75–1.26)	0.90 (0.67–1.19)
Prostate Number of cases/controls	2507/30596	1473/17901	476/4891	2461/30769	749/9443	616/7864
Multivariable-adjusted HR (95% CI)	1.00 (Referent)	0.98 (0.92–1.04)	1.05 (0.94–1.17)	0.98 (0.93–1.03)	0.97 (0.89–1.05)	0.97 (0.89–1.06)
Thyroid Number of cases/controls	110/32993	69/19305	22/5345	122/33108	31/10161	26/8454
Multivariable-adjusted HR (95% CI)	1.00 (Referent)	1.09 (0.81–1.48)	1.35 (0.80–2.27)	1.12 (0.86–1.44)	0.94 (0.63–1.40)	0.92 (0.60–1.41)
Upper Gastrointestinal Tract Number of cases/controls	204/32899	100/19274	44/5323	182/33048	74/10118	42/8438
Multivariable-adjusted HR (95% CI)	1.00 (Referent)	0.80 (0.63–1.02)	1.08 (0.74–1.58)	0.89 (0.73–1.09)	1.12 (0.85–1.47)	0.81 (0.58–1.13)
Esophagus Number of cases/controls	90/32899	43/19274	11/5323	81/33048	29/10118	17/8438
Multivariable-adjusted HR (95% CI)	1.00 (Referent)	0.82 (0.57–1.19)	0.78 (0.39–1.56)	0.90 (0.67–1.22)	1.08 (0.71–1.65)	0.75 (0.45–1.26)
Stomach Number of cases/controls	114/32899	57/19274	33/5323	101/33048	45/10118	25/8438
Multivariable-adjusted HR (95% CI)	1.00 (Referent)	0.79 (0.57–1.09)	1.25 (0.79–1.98)	0.88 (0.67–1.15)	1.15 (0.81–1.63)	0.85 (0.55–1.31)

^a^ Adjusted for age at randomization (continuous), race (non-Hispanic white, non-Hispanic black, all others combined), sex, body mass index (continuous), smoking status (never, current, former), history of diabetes (yes/no), family history of cancer (yes/no), latitude of study center (<34°N, 34-<42°N, >42°N)

^b^ lung cancer model additionally adjusted for cigar smoking (yes/no), pipe smoking (yes/no), and packyears of cigarette smoking (quartiles)

## Discussion

Based on data from 3,795 cancer cases and 3,856 controls in the PLCO Trial, we observed significant effect modification of the circulating vitamin D-cancer risk association by Gc isoform (Gc1-2 and Gc2-2 vs. Gc1-1) for bladder cancer, but not for other cancer sites, including colorectal and lung cancer, for which prior studies have noted interactions [15, 18], or for all cancers combined. Furthermore, with a few exceptions, we did not observe associations between the six Gc isoforms and cancer risk among 109,746 participants with *GC* genetic data.

We have previously noted in PLCO effect modification by Gc isoforms of the associations between circulating 25(OH)D and overall and lung cancer survival, whereby the benefit from higher vitamin D was only evident for cases with the Gc2 isoform [15] (HR = 0.38 for overall cancer mortality for those with the Gc2-2 isoform vs. 0.94 for those with the Gc1-1 isoform, and HR = 0.30 for lung cancer mortality for those with either the Gc1-2 or Gc2-2 isoform vs. 0.95 for those with the Gc1-1 isoform). Factors that influence the risk of developing cancer can differ from those impacting survival. For example, smoking is associated with a lower risk of prostate cancer but also adversely impacts prostate cancer survival [36]. Similarly, higher vitamin D status is associated with elevated prostate cancer risk [10] but with improved prostate cancer survival [37], with the latter difference not being due to detection or collider biases [38]. The opposite outcomes for risk and survival in our PLCO analyses could also suggest differential inhibitory effects of vitamin D/Gc isoform on tumor initiation vs. growth, invasion, or metastasis via vitamin D receptor (VDR) response elements. For example, there could be a greater impact on genes regulated by VDR related to angiogenesis, invasion, and metastasis (e.g., *HIF1α* and *IL-8*) [39] compared with genes that influence tumor initiation (i.e., malignant transformation) (e.g. *MYC* and *RB*) [39].

Others have previously noted effect modification by Gc isoforms of the associations between circulating 25(OH)D and cancer risk or survival. Gibbs *et al*. found a similar genetic effect modification pattern for 25(OH)D and colorectal adenoma and colorectal cancer risk, and colorectal cancer mortality outcomes [16–18], as well as for vitamin D supplementation (1000 IU/day) and colorectal adenoma recurrence [19]. For example, the HR for colorectal cancer mortality for deficient vs. sufficient circulating vitamin D was 2.24 for cases with the Gc2 isoform and 0.94 among cases without the Gc2 isoform (p-interaction = 0.0002) [16]. Similarly, in the Danish Diet, Cancer, and Health prospective cohort study, the reduced risk for higher vitamin D intake and colorectal cancer was only noted among participants with the Gc2 isoform (IRR for 3 ug/day increase in vitamin D intake = 0.90 for Gc2-2 vs. 0.99 for Gc1-1), while the isoform itself was not associated with colorectal cancer [20]. In contrast, data from the UK Biobank showed reduced colorectal cancer risk with higher serum 25(OH)D concentration but no significant interaction with the Gc isoform [21], and an analysis of the Nurses’ Health Study and Health Professionals Follow-up Study did not find effect modification of the vitamin D-colorectal cancer association by Gc isoform [22]. Finally, a population-based case-control study in Germany showed an inverse association for breast cancer risk for women with the Gc2-2 isoform compared with the Gc1s-1s isoform, but no interaction with 25(OH)D [12]. Differences in the observed effect modification across studies may be due to population differences, with participants being drawn from different countries, having different races/ethnicities, sex, and likely other risk factors such as smoking, physical activity, anthropometry, or diets. Such differences may be relevant as vitamin D binding protein concentrations have been associated with factors such as oral contraceptive use, BMI, long-term smoking, and triglyceride and cholesterol levels [40]. In addition, participants in the various studies may have different ranges of low vs. adequate circulating vitamin D.

With the exception of bladder cancer, we found no significant effect modification of the 25(OH)D-cancer risk association by Gc isoforms, and although we did observe reduced colorectal cancer risk for higher 25(OH)D, there was no interaction with *GC*. In contrast, individuals with higher 25(OH)D and any Gc2 isoform had a better lung cancer outcome (i.e., no increased risk) compared with individuals with only Gc1 isoforms (higher risk), and even though the interaction was not statistically significant, it was similar in direction to our previous finding of improved lung cancer survival in cases with higher 25(OH)D and any Gc2 isoform [15]. Regarding bladder cancer, we observed inverse vitamin D risk associations in participants with any Gc2 isoform (Gc1-2 or Gc2) but not in those with the Gc1-1 isoform. Why this effect modification might exist for bladder cancer, but not other cancers, can only be speculated. For example, genes related to inflammation (e.g. *COX2*) [39] could be particularly affected by the interaction between vitamin D and Gc isoform. Alternatively, the finding could be due to chance and should be examined in additional studies.

For the genotype-only examination of *GC* and cancer risk, we noted increased risks of biliary tract and male breast cancer for individuals with various Gc isoforms, but these were based on very small case numbers. We also observed reduced risk for melanoma for individuals with the Gc1f-Gc1s and Gc1f-Gc1f isoforms, findings consistent with data from a pooled analysis of two previous studies that encompassed 4,490 cases diagnosed in Australia, Canada, Italy, and the United States that showed lower melanoma-specific death among cases with any Gc1f isoform vs. no Gc1f (HR = 0.63, 95% CI 0.47–0.83) [41].

Our study has strengths and limitations. It is among the largest prospective studies to examine effect modification by Gc isoforms on the association between vitamin D and cancer risk. The analyses among the nested case-control sets with vitamin D biomarker data included nearly 3,800 cases and over 7,600 individuals, while the genetic-only analysis included over 26,700 cases among nearly 110,000 participants. Even with these sample sizes, however, there was limited power to detect associations within strata of Gc isoform for uncommon and rare cancers, and the number of statistical tests that were run could have increased the type one error rate. The PLCO population consists of mainly White individuals, which limited our ability to examine associations in other racial and ethnic groups and limited the generalizability of our findings to those populations. This is particularly relevant because circulating 25(OH)D concentrations and Gc isoforms differ by race and could be related to disparate vitamin D/cancer incidence and survival associations by race [42, 43]. Circulating 25(OH)D measurements in PLCO were calibrated to a standard assay within an international pooling project of 17 cohorts and empirically were at the median of all the cohorts combined (median, 10-90^th^ percentiles in PLCO = 55 (29–87) and for all 17 cohorts = 56 (31–85). All cancers were prospectively diagnosed, which minimized reverse causality. Although vitamin D concentrations vary by season, we accounted for this using season-specific 25(OH)D quantiles. We also created quantiles separately for each nested case-control set because the vitamin D assays for these sets were conducted at laboratories using different methods. We adjusted our models for several covariates that could potentially be associated with cancer risk, but the possibility of residual confounding cannot be eliminated. While vitamin D was assayed in blood samples from single time points, a common limitation of most observational studies, methodological studies have demonstrated 25(OH)D concentrations to be moderately correlated over 14 years [44–47] and to be stable up to 30 years [48, 49].

## Conclusions

Gc isoforms of the vitamin D binding protein did not modify the association between circulating vitamin D and cancer risk within the PLCO Trial, either for overall cancer or specific cancer sites such as lung, colorectum, breast and prostate, although an interaction was noted for bladder cancer. Gc isoforms alone were not significantly associated with overall or site-specific cancer, with the possible exceptions of increased risk of biliary tract and male breast cancer for individuals with the Gc2-Gc2 isoform and the Gc1f-Gc1s or Gc1f-Gc1f isoforms, respectively, and a reduced risk of melanoma for individuals with the Gc1f-Gc1s or Gc1f-Gc1f isoforms. Additional large studies that include more racially and ethnically diverse populations are needed.

## Supporting information

S1 Table Cross tabulation of rs4588 and rs7041 to define the vitamin D binding protein Gc groups. (DOCX)

S2 Table Serum 25(OH)D quintile cut-points based on the controls, stratified by cancer site, season, and sex. (DOCX)

S3 Table Serum 25(OH)D tertile cutpoints based on the controls, stratified by cancer site, season, and sex. (DOCX)

S4 Table Distribution of Gc isoform by race and ethnicity. (DOCX)

S5 Table Selected baseline characteristics of cases and non-cases with *GC* SNP data. (DOCX)

S6 Table Association between Gc isoform and overall and organ-specific cancer risk in 4,408 black individuals. (DOCX)
