# Supplementary material for: Vitamin D binding protein genetic isoforms, serum vitamin D, and cancer risk in the Prostate, Lung, Colorectal, and Ovarian (PLCO) Cancer Screening Trial
Source: PLoS One. 2024 Dec 20;19(12):e0315252. doi: 10.1371/journal.pone.0315252 (PMC11661580; doi:10.1371/journal.pone.0315252)
Supplement: S1 Table — (DOCX) [file pone.0315252.s001.docx]

**S1 Table. Cross tabulation of rs4588 and rs7041 to define the vitamin D binding protein Gc groups^a^**

|  | | rs7041 | | | Total Gc1 and Gc2 | |
| --- | --- | --- | --- | --- | --- | --- |
|  |  | Glu/Glu | Asp/Glu | Asp/Asp |  |  |
| rs4588 | Thr/Thr | Gc1s-Gc1s  n=33,103  30.16% | Gc1f-Gc1s  n=19,374  17.65% | Gc1f-Gc1f  n=5,367  4.89% | Total Gc1-1  n=57,844  52.71% |  |
|  | Thr/Lys |  | Gc1s-Gc2 n=33,230  30.28% | Gc1f-Gc2  n=10,192  9.29% | Total Gc1-2  n= 43,422  39.57% | Any Gc2  n=51,902  47.29% |
|  | Lys/Lys |  |  | Gc2-Gc2  n=8,480  7.73% | Total Gc2-2  n= 8,480  7.73% |  |

^a^ Cross tabulations as defined by Abbas, et al. [12]. Asp, aspartic acid; Gc, group-specific component; Glu, glutamic acid; Lys, lysine; Thr, threonine
