# Supplementary material for: Vitamin D binding protein genetic isoforms, serum vitamin D, and cancer risk in the Prostate, Lung, Colorectal, and Ovarian (PLCO) Cancer Screening Trial
Source: PLoS One. 2024 Dec 20;19(12):e0315252. doi: 10.1371/journal.pone.0315252 (PMC11661580; doi:10.1371/journal.pone.0315252)
Supplement: S2 Table — (DOCX) [file pone.0315252.s002.docx]

**S2 Table. Serum 25(OH)D quintile cut-points based on the controls, stratified by cancer site, season^a^, and sex**

| Cancer site | Quintile of serum 25(OH)D (nmol/L) | | | | |
| --- | --- | --- | --- | --- | --- |
|  | Q1 | Q2 | Q3 | Q4 | Q5 |
| Bladder |  |  |  |  |  |
| Summer, Males | < 47.7 | >47.7- < 56.9 | >56.9 - < 64.1 | >64.1 - < 76.3 | >76.3 |
| Summer, Females | < 35.4 | >35.4- < 43.4 | >43.4 - < 53.4 | >53.4 - < 61.4 | >61.4 |
| Winter, Males | < 32.4 | >32.4- < 40.5 | >40.5 - < 51.8 | >51.8 - < 67.8 | >67.8 |
| Winter, Females | < 30.1 | >30.1- < 41.9 | >41.9 - < 50.5 | >50.5 - < 63.4 | >63.4 |
| Breast |  |  |  |  |  |
| Summer, Females | < 51.7 | >51.7- < 63.6 | >63.6 - < 74.6 | >74.6 - < 87.6 | >87.6 |
| Winter, Females | < 40.2 | >40.2- < 52.2 | >52.2 - < 63.5 | >63.5 - < 78.6 | >78.6 |
| Colorectum |  |  |  |  |  |
| Summer, Males | < 47.7 | >47.7- < 61.4 | >61.4 - < 71.9 | >71.9 - < 83.1 | >83.1 |
| Summer, Females | < 40.2 | >40.2- < 53.2 | >53.2 - < 60.4 | >60.4 - < 73.6 | >73.6 |
| Winter, Males | < 34.2 | >34.2- < 45.4 | >45.4 - < 56.7 | >56.7 - < 68.0 | >68.0 |
| Winter, Females | < 30.1 | >30.1- < 42.4 | >42.4 - < 53.8 | >53.8 - < 68.9 | >68.9 |
| Endometrium |  |  |  |  |  |
| Summer, Females | < 40.3 | >40.3- < 49.8 | >49.9- < 63.4 | >63.4- < 71.6 | >71.6 |
| Winter, Females | < 29.2 | >29.2- < 36.9 | >36.9- < 51.9 | >51.9- < 62.4 | >62.4 |
| Hematopoietic |  |  |  |  |  |
| Summer, Males | < 43.4 | >43.4- < 53.7 | >53.7 - < 58.7 | >58.7 - < 73.1 | >73.1 |
| Summer, Females | < 35.7 | >35.7- < 55.2 | >55.2 - < 61.9 | >61.9 - < 75.6 | >75.6 |
| Winter, Males | < 36.9 | >36.9- < 55.2 | >55.2 - < 61.9 | >61.9 - < 75.6 | >75.6 |
| Winter, Females | < 27.7 | >27.7- < 37.7 | >37.7 - < 48.7 | >48.7 - < 64.4 | >64.4 |
| Kidney |  |  |  |  |  |
| Summer, Males | < 50.9 | >50.9- < 57.9 | >57.9- < 68.6 | >68.6- < 79.9 | >79.9 |
| Summer, Females | < 38.3 | >38.3- < 51.8 | >51.8- < 63.3 | >63.3 < 73.8 | >73.8 |
| Winter, Males | < 40.4 | >40.4- < 51.2 | >51.2- < 57.7 | >57.7- < 65.9 | >65.9 |
| Winter, Females | < 27.2 | >27.2- < 39.2 | >39.2- < 43.4 | >43.4- < 52.7 | >52.7 |
| Lung |  |  |  |  |  |
| Summer, Males | < 49.8 | >49.8- < 63.7 | >63.7 - < 74.8 | >74.8 - < 92.7 | >92.7 |
| Summer, Females | < 46.0 | >46.0- < 62.0 | >62.0 - < 81.2 | >81.2 - < 94.4 | >94.4 |
| Winter, Males | < 38.4 | >38.4- < 49.3 | >49.3 - < 58.9 | >58.9 - < 74.0 | >74.0 |
| Winter, Females | < 45.2 | >45.2- < 56.3 | >56.3 - < 68.2 | >68.2 - < 86.9 | >86.9 |
| Ovary |  |  |  |  |  |
| Summer, Females | < 40.4 | >40.4- < 49.9 | >49.9- < 56.9 | >56.9- < 67.9 | >67.9 |
| Winter, Females | < 37.7 | >37.7- < 45.4 | >45.4- < 49.2 | >49.2- < 56.9 | >56.9 |
| Pancreas (set 1) |  |  |  |  |  |
| Summer, Males | < 60.4 | >60.4- < 69.6 | >69.6 - < 78.6 | >78.6- < 88.8 | >88.8 |
| Summer, Females | < 46.0 | >46.0- < 61.5 | >61.5 - < 74.5 | >74.5- < 84.3 | >84.3 |
| Winter, Males | < 39.8 | >39.8- < 49.1 | >49.1 - < 61.0 | >61.0- < 71.7 | >71.7 |
| Winter, Females | < 27.1 | >27.1- < 51.8 | >51.8 - < 62.6 | >62.6- < 70.3 | >70.3 |
| Pancreas (set 2) |  |  |  |  |  |
| Summer, Males | < 51.4 | >51.4- < 58.9 | >58.9 - < 80.2 | >80.2- < 93.2 | >93.2 |
| Summer, Females | < 35.5 | >35.5- < 54.3 | >54.3 - < 63.2 | >63.2- < 73.3 | >73.3 |
| Winter, Males | < 43.6 | >43.6- < 48.0 | >48.0 - < 50.7 | >50.7- < 62.5 | >62.5 |
| Winter, Females | < 25.2 | >25.2- < 46.8 | >46.8 - < 59.3 | >59.3- < 69.5 | >69.5 |
| Prostate (White individuals) |  |  |  |  |  |
| Summer, Males | < 46.4 | >46.4- < 55.4 | >55.4 - < 65.1 | >65.1 - < 76.4 | >76.4 |
| Winter, Males | < 36.7 | >36.7- < 45.7 | >45.7 - < 54.2 | >54.2 - < 66.4 | >66.4 |
| Prostate (Black individuals) |  |  |  |  |  |
| Summer, Males | < 34.7 | >34.7- < 47.2 | >47.2 - < 58.4 | >58.4- < 76.7 | >76.4 |
| Winter, Males | < 26.2 | >26.2- < 37.9 | >37.9 - < 46.9 | >46.9- < 60.4 | >60.4 |
| Upper gastrointestinal tract |  |  |  |  |  |
| Summer, Males | < 50.8 | >50.8- < 62.9 | >62.9- < 70.6 | >70.6- < 82.7 | >82.7 |
| Summer, Females | < 28.0 | >28.0- < 30.0 | >30.0- < 38.4 | >38.4- < 39.7 | >39.7 |
| Winter, Males | < 33.7 | >33.7- < 44.7 | >44.7- < 49.7 | >49.7- < 63.6 | >63.6 |
| Winter, Females | < 33.7 | >33.7- < 34.4 | >34.4- < 46.7 | >46.7- < 62.4 | >62.4 |

25(OH)D, 25-hydroxyvitamin D; PLCO, Prostate, Lung, Colorectal, and Ovarian Cancer Screening Trial; Q, quantile

^a^ Winter is defined as December-May, Summer is defined as June-November.
