# Supplementary material for: Vitamin D binding protein genetic isoforms, serum vitamin D, and cancer risk in the Prostate, Lung, Colorectal, and Ovarian (PLCO) Cancer Screening Trial
Source: PLoS One. 2024 Dec 20;19(12):e0315252. doi: 10.1371/journal.pone.0315252 (PMC11661580; doi:10.1371/journal.pone.0315252)
Supplement: S3 Table — (DOCX) [file pone.0315252.s003.docx]

**S3 Table. Serum 25(OH)D tertile cutpoints based on the controls, stratified by cancer site, season^a^, and sex**

| Cancer site | Tertile of serum 25(OH)D (nmol/L) | | |
| --- | --- | --- | --- |
|  | Q1 | Q2 | Q3 |
| Bladder |  |  |  |
| Summer, Males | < 54.5 | >54.5 - < 70.1 | >70.1 |
| Summer, Females | < 43.1 | >43.1 - < 55.7 | >55.7 |
| Winter, Males | < 38.1 | >38.1 - < 55.3 | >55.3 |
| Winter, Females | < 34.6 | >34.6 - < 60.3 | >60.3 |
| Breast |  |  |  |
| Summer, Females | < 60.2 | >60.2 - < 78.6 | >78.6 |
| Winter, Females | < 48.7 | >48.7 - < 68.6 | >68.6 |
| Colorectum |  |  |  |
| Summer, Males | < 58.0 | >58.0 - < 74.9 | >74.9 |
| Summer, Females | < 48.8 | >48.8 - < 64.6 | >64.6 |
| Winter, Males | < 43.1 | >43.1 - < 59.7 | >59.7 |
| Winter, Females | < 38.3 | >38.3 - < 57.2 | >57.2 |
| Endometrium |  |  |  |
| Summer, Females | < 47.4 | >47.4- < 65.4 | >65.4 |
| Winter, Females | < 35.4 | >35.4- < 57.2 | >57.2 |
| Hematopoietic |  |  |  |
| Summer, Males | < 51.0 | >51.0 - < 61.2 | >61.2 |
| Summer, Females | < 45.7 | >45.7 - < 65.4 | >65.4 |
| Winter, Males | < 43.2 | >43.2 - < 59.9 | >59.9 |
| Winter, Females | < 33.2 | >33.2 - < 49.9 | >49.9 |
| Kidney |  |  |  |
| Summer, Males | < 56.4 | >56.4- < 70.9 | >70.9 |
| Summer, Females | < 48.2 | >48.2- < 66.9 | >66.9 |
| Winter, Males | < 45.2 | >45.2- < 59.2 | >59.2 |
| Winter, Females | < 31.2 | >31.2- < 49.2 | >49.2 |
| Lung |  |  |  |
| Summer, Males | < 59.9 | >59.9 - < 79.6 | >79.6 |
| Summer, Females | < 56.0 | >56.0 - < 84.3 | >84.3 |
| Winter, Males | < 46.6 | >46.6 - < 62.5 | >62.5 |
| Winter, Females | < 51.8 | >51.8 - < 75.8 | >75.8 |
| Ovary |  |  |  |
| Summer, Females | < 46.9 | >46.9- < 59.2 | >59.2 |
| Winter, Females | < 43.2 | >43.2- < 54.4 | >54.4 |
| Pancreas (set 1) |  |  |  |
| Summer, Males | < 66.2 | >66.2 - < 80.4 | >80.4 |
| Summer, Females | < 59.8 | >59.8 - < 81.3 | >81.3 |
| Winter, Males | < 46.7 | >46.7 - < 66.1 | >66.1 |
| Winter, Females | < 49.5 | >49.5 - < 65.3 | >65.3 |
| Pancreas (set 2) |  |  |  |
| Summer, Males | < 54.5 | >54.5 - < 89.9 | >89.9 |
| Summer, Females | < 44.3 | >44.3 - < 71.4 | >71.4 |
| Winter, Males | < 48.0 | >48.0 - < 56.7 | >56.7 |
| Winter, Females | < 45.6 | >45.6 - < 68.9 | >68.9 |
| Prostate (White individuals) |  |  |  |
| Summer, Males | < 52.7 | >52.7 - < 69.1 | >69.1 |
| Winter, Males | < 43.7 | >43.7 - < 57.2 | >57.2 |
| Prostate (Black individuals) |  |  |  |
| Summer, Males | < 42.2 | >42.2 - < 61.9 | >61.9 |
| Winter, Males | < 32.7 | >32.7 - < 51.4 | >51.4 |
| Upper gastrointestinal tract |  |  |  |
| Summer, Males | < 60.7 | >60.7- < 74.6 | >74.6 |
| Summer, Females | < 28.0 | >28.0- < 39.7 | >39.7 |
| Winter, Males | < 41.2 | >41.2- < 51.4 | >51.4 |
| Winter, Females | < 34.4 | >34.4- < 46.7 | >46.7 |

25(OH)D, 25-hydroxyvitamin D; PLCO, Prostate, Lung, Colorectal, and Ovarian Cancer Screening Trial; Q, quantile

^a^ Winter is defined as December-May, Summer is defined as June-November
