# Supplementary material for: Vitamin D binding protein genetic isoforms, serum vitamin D, and cancer risk in the Prostate, Lung, Colorectal, and Ovarian (PLCO) Cancer Screening Trial
Source: PLoS One. 2024 Dec 20;19(12):e0315252. doi: 10.1371/journal.pone.0315252 (PMC11661580; doi:10.1371/journal.pone.0315252)
Supplement: S4 Table — (DOCX) [file pone.0315252.s004.docx]

**S4 Table. Distribution of Gc isoform by race and ethnicity^a^**

| Characteristic | Gc1s-Gc1s | Gc1f-Gc1s | Gc1f-Gc1f | Gc1s-Gc2 | Gc1f-Gc2 | Gc2-Gc2 |
| --- | --- | --- | --- | --- | --- | --- |
| American Indian individuals | 63 (26.7) | 50 (21.2) | 17 (7.2) | 55 (23.3) | 31 (13.1) | 20 (8.5) |
| Asian individuals | 266 (8.5) | 726 (23.2) | 673 (21.5) | 474 (15.1) | 768 (24.5) | 225 (7.2) |
| Black individuals, non-Hispanic | 138 (3.1) | 1,075 (24.4) | 2,235 (50.7) | 190 (4.3) | 710 (16.1) | 60 (1.4) |
| Hispanic individuals | 458 (27.5) | 410 (24.6) | 107 (6.4) | 390 (23.4) | 193 (11.6) | 106 (6.4) |
| Pacific Islander individuals | 25 (8.1) | 81 (26.2) | 58 (18.8) | 61 (19.7) | 60 (19.4) | 24 (7.8) |
| White individuals, non-Hispanic | 31,582 (32.2) | 16,702 (17.0) | 2,196 (2.2) | 31,541(32.1) | 8,266 (8.4) | 7,921 (8.1) |
| Missing | 571 (31.9) | 330 (18.5) | 81 (4.5) | 519 (29.0) | 164 (9.2) | 124 (6.9) |

^a^values are number (row percent)
