# Supplementary material for: Vitamin D binding protein genetic isoforms, serum vitamin D, and cancer risk in the Prostate, Lung, Colorectal, and Ovarian (PLCO) Cancer Screening Trial
Source: PLoS One. 2024 Dec 20;19(12):e0315252. doi: 10.1371/journal.pone.0315252 (PMC11661580; doi:10.1371/journal.pone.0315252)
Supplement: S5 Table — (DOCX) [file pone.0315252.s005.docx]

**S5 Table. Selected baseline characteristics of cases and non-cases with *GC* SNP data**

| Characteristic | Cancer cases  (n=26,713) | Non-Cases  (n=83,033) | *P* ^a^ |
| --- | --- | --- | --- |
| Age at randomization, years (median, 10^th^-90^th^) | 62 (56-70) | 61 (55-70) | <0.0001 |
| Sex, No. (%) |  |  | <0.0001 |
| Female | 10,590 (39.6) | 45,869 (55.2) |  |
| Male | 16,123 (60.4) | 37,164 (44.8) |  |
| Race, No. (%) |  |  | <0.0001 |
| American Indian individuals | 40 (0.2) | 196 (0.2) |  |
| Asian individuals | 655 (2.5) | 2,477 (3.0) |  |
| Black individuals, non-Hispanic | 1,119 (4.2) | 3,289 (4.0) |  |
| Hispanic individuals | 333 (1.3) | 1,331 (1.6) |  |
| Pacific Islander individuals | 80 (0.3) | 229 (0.3) |  |
| White individuals, non-Hispanic | 24,024 (89.9) | 74,184 (89.3) |  |
| missing | 462 (1.7) | 1,327 (1.6) |  |
| BMI, kg/m^2^ (median, 10^th^-90^th^) | 26.6 (22.3-33.1) | 26.6 (21.9-33.4) | <0.0001 |
| Smoking status, No. (%) |  |  | <0.0001 |
| Never | 10,879 (40.7) | 40,734 (49.1) |  |
| Former | 12,133 (45.4) | 34,322 (41.3) |  |
| Current | 3,238 (12.1) | 6,671 (8.0) |  |
| Missing | 463 (1.7) | 1,306 (1.6) |  |
| Vigorous physical activity, No. (%)^b^ |  |  | 0.94 |
| None or <1 hours/week | 4,533 (33.2) | 13,733 (33.1) |  |
| 1-2 hours/week | 3,783 (27.7) | 11,545 (27.8) |  |
| 3+ hours/week | 5,347 (39.1) | 16,195 (39.1) |  |
| History of diabetes, No. (% yes) | 1,746 (6.5) | 5,391 (6.5) | 0.09 |
| Family history of cancer, No. (% yes)^c^ | 15,201 (56.9) | 45,578 (54.9) | <0.0001 |
| Latitude of study center, No. (%) |  |  | <0.0001 |
| <34^o^N | 2,015 (7.5) | 7,137 (8.6) |  |
| 34-<42^o^N | 11,746 (44.0) | 37,598 (45.3) |  |
| *>*42^o^N | 12,952 (48.5) | 38,298 (46.1) |  |
| Gc isoform, No. (%) |  |  | 0.31 |
| Gc1s-Gc1s | 8,199 (30.7) | 24,904 (30.0) |  |
| Gc1f-Gc1s | 4,688 (17.6) | 14,686 (17.7) |  |
| Gc1f-Gc1f | 1,311 (4.9) | 4,056 (4.9) |  |
| Gc1s-Gc2 | 8,056 (30.2) | 25,174 (30.3) |  |
| Gc1f-Gc2 | 2,442 (9.1) | 7,750 (9.3) |  |
| Gc2-Gc2 | 2,017 (7.7) | 6,463 (7.8) |  |

BMI = body mass index; 25(OH)D, 25-hydroxyvitamin D

^a^ *P*-values based on 2-sided Wilcoxon rank sum tests for continuous variables and 2-sided chi-square tests for categorical variables

^b^ Only available for approximately half of the population as this was only ascertained in the screening arm

^c^ Family history of any cancer reported in first degree relatives (i.e., parents, full-siblings, and children)
