# Supplementary material for: Vitamin D binding protein genetic isoforms, serum vitamin D, and cancer risk in the Prostate, Lung, Colorectal, and Ovarian (PLCO) Cancer Screening Trial
Source: PLoS One. 2024 Dec 20;19(12):e0315252. doi: 10.1371/journal.pone.0315252 (PMC11661580; doi:10.1371/journal.pone.0315252)
Supplement: S6 Table — (DOCX) [file pone.0315252.s006.docx]

**S6 Table. Association between Gc isoform and overall and organ-specific cancer risk in 4,408 black individuals^a^**

| Cancer site | Gc Isoform | | | | | |
| --- | --- | --- | --- | --- | --- | --- |
|  | Gc1s-Gc1s | Gc1f-Gc1s | Gc1f-Gc1f | Gc1s-Gc2 | Gc1f-Gc2 | Gc2-Gc2 |
| Overall  Number of cases/controls | 36/102 | 276/799 | 573/1662 | 42/148 | 178/532 | 14/46 |
| Multivariable-adjusted HR (95% CI) | 1.00 (Referent) | 1.03 (0.72-1.45) | 1.03 (0.74-1.45) | 0.84 (0.52-1.32) | 1.02 (0.71-1.45) | 0.87 (0.47-1.62) |
| Biliary tract  Number of cases/controls | 0/138 | 2/1073 | 3/2232 | 0/190 | 2/708 | 0/60 |
| Multivariable-adjusted HR (95% CI)  Unstable, not calculated |  |  |  |  |  |  |
| Bladder  Number of cases/controls | 2/136 | 7/1068 | 9/2226 | 1/189 | 10/700 | 1/59 |
| Multivariable-adjusted HR (95% CI) | 1.00 (Referent) | 0.47 (0.10-2.26) | 0.29 (0.06-1.36) | 0.37 (0.03-4.13) | 1.04 (0.23-4.78) | 1.17 (0.11-12.98) |
| Breast (female)  Number of cases/controls | 7/131 | 24/1051 | 59/2176 | 5/185 | 30/680 | 2/58 |
| Multivariable-adjusted HR (95% CI) | 1.00 (Referent) | 0.46 (0.20-1.07) | 0.54 (0.25-1.20) | 0.47 (0.15-1.50) | 0.80 (0.35-1.84) | 0.63 (0.13-3.03) |
| Breast (male)  Number of cases/controls | 0/138 | 2/1073 | 1/2234 | 0/190 | 0/710 | 0/60 |
| Multivariable-adjusted HR (95% CI)  Unstable, not calculated |  |  |  |  |  |  |
| Colorectum  Number of cases/controls | 3/135 | 31/1044 | 53/2182 | 3/187 | 15/695 | 5/55 |
| Multivariable-adjusted HR (95% CI) | 1.00 (Referent) | 1.33 (0.41-4.36) | 1.09 (0.34-3.49) | 0.70 (0.14-3.48) | 0.98 (0.28-3.39) | 3.82 (0.91-15.99) |
| Colon  Number of cases/controls | 3/135 | 24/1044 | 47/2182 | 2/187 | 15/695 | 4/55 |
| Multivariable-adjusted HR (95% CI) | 1.00 (Referent) | 1.04 (0.31-3.42) | 0.97 (0.30-2.83) | 0.47 (0.08-2.83) | 0.98 (0.28-3.40) | 3.09 (0.69-13.82) |
| Rectum  Number of cases/controls | 0/135 | 5/1044 | 3/2182 | 1/187 | 0/695 | 1/55 |
| Multivariable-adjusted HR (95% CI)  Unstable, not calculated |  |  |  |  |  |  |
| Endometrium  Number of cases/controls | 0/138 | 3/1072 | 8/2227 | 1/189 | 2/708 | 0/60 |
| Multivariable-adjusted HR (95% CI)  Unstable, not calculated |  |  |  |  |  |  |
| Glioma  Number of cases/controls | 0/138 | 2/1073 | 2/2233 | 1/189 | 2/7008 | 0/60 |
| Multivariable-adjusted HR (95% CI)  Unstable, not calculated |  |  |  |  |  |  |
| Hematopoietic  Number of cases/controls | 2/136 | 29/1046 | 43/2192 | 5/185 | 15/695 | 0/60 |
| Multivariable-adjusted HR (95% CI) | 1.00 (Referent) | 1.96 (0.47-8.24) | 1.43 (0.35-5.90) | 1.81 (0.35-9.32) | 1.55 (0.35-6.79) |  |
| Head and Neck  Number of cases/controls | 1/137 | 6/1069 | 11/2224 | 1/189 | 5/705 | 0/60 |
| Multivariable-adjusted HR (95% CI) | 1.00 (Referent) | 0.75 (0.09-6.36) | 0.69 (0.09-5.38) | 0.70 (0.04-11.24) | 1.00 (0.12-8.62) |  |
| Kidney  Number of cases/controls | 2/136 | 9/1066 | 28/2207 | 2/188 | 8/702 | 1/59 |
| Multivariable-adjusted HR (95% CI) | 1.00 (Referent) | 0.59 (0.13-2.74) | 0.91 (0.22-3.85) | 0.71 (0.10-5.04) | 0.83 (0.18-3.89) | 1.16 (0.10-12.80) |
| Liver  Number of cases/controls | 1/137 | 6/1069 | 8/2227 | 0/190 | 2/708 | 1/59 |
| Multivariable-adjusted HR (95% CI) | 1.00 (Referent) | 0.67 (0.08-5.63) | 0.43 (0.05-3.45) |  | 0.35 (0.03-3.85) | 1.91 (0.12-30.93) |
| Lung ^b^  Number of cases/controls | 5/133 | 61/1014 | 107/2128 | 9/181 | 36/674 | 1/59 |
| Multivariable-adjusted HR (95% CI) | 1.00 (Referent) | 1.65 (0.66-4.15) | 1.41 (0.57-3.49) | 1.24 (0.41-3.73) | 1.58 (0.62-4.06) | 0.45 (0.05-3.85) |
| Melanoma  Number of cases/controls | 0/138 | 1/1074 | 1/2234 | 1/189 | 1/709 | 0/60 |
| Multivariable-adjusted HR (95% CI)  Unstable, not calculated |  |  |  |  |  |  |
| Ovary  Number of cases/controls | 0/138 | 5/1070 | 11/2224 | 1/189 | 0/710 | 0/60 |
| Multivariable-adjusted HR (95% CI)  Unstable, not calculated |  |  |  |  |  |  |
| Pancreas  Number of cases/controls | 0/138 | 9/1066 | 23/2212 | 2/188 | 11/699 | 0/60 |
| Multivariable-adjusted HR (95% CI)  Unstable, not calculated |  |  |  |  |  |  |
| Prostate  Number of cases/controls | 13/125 | 104/971 | 239/1996 | 17/173 | 60/650 | 4/56 |
| Multivariable-adjusted HR (95% CI) | 1.00 (Referent) | 0.99 (0.55-1.76) | 1.15 (0.66-2.02) | 0.98 (0.48-2.02) | 0.96 (0.53-1.76) | 0.61 (0.20-1.88) |
| Thyroid  Number of cases/controls | 1/137 | 5/1070 | 8/2227 | 0/190 | 1/709 | 1/59 |
| Multivariable-adjusted HR (95% CI) | 1.00 (Referent) | 0.67 (0.08-5.74) | 0.50 (0.06-4.07) |  | 0.19 (0.01-2.99) | 2.32 (0.14-37.35) |
| Upper Gastrointestinal Tract  Number of cases/controls | 0/138 | 9/1066 | 22/2213 | 0/190 | 5/705 | 1/59 |
| Multivariable-adjusted HR (95% CI)  Unstable, not calculated |  |  |  |  |  |  |
| Esophagus  Number of cases/controls | 0/138 | 2/1066 | 4/2213 | 0/190 | 3/705 | 1/59 |
| Multivariable-adjusted HR (95% CI)  Unstable, not calculated |  |  |  |  |  |  |
| Stomach  Number of cases/controls | 0/138 | 7/1066 | 18/2213 | 0/190 | 2/705 | 0/59 |
| Multivariable-adjusted HR (95% CI)  Unstable, not calculated |  |  |  |  |  |  |

^a^ Adjusted for age at randomization (continuous), sex, body mass index (continuous), smoking status (never, current, former), history of diabetes (yes/no), family history of cancer (yes/no), latitude of study center (<34^o^N, 34-<42^o^N, >42^o^N)

^b^ lung cancer model additionally adjusted for cigar smoking (yes/no), pipe smoking (yes/no), and packyears of cigarette smoking (quartiles)
